# Epidemiological Surveys of Yam Fields in Côte d’Ivoire Revealed the First Detection of YMMV and Evidence of Episomal Badnavirus

**DOI:** 10.3390/v17121586

**Published:** 2025-12-05

**Authors:** Maïmouna M. Koné, Justin S. Pita, Cyrielle Ndougonna, Daniel H. Otron, Mariam Combala, Guy R. Eboulem, William J.-L. Amoakon, Bekanvié S. M. Kouakou, Angela O. Eni, Fatogoma Sorho, Fidèle Tiendrébéogo

**Affiliations:** 1 Laboratoire d’Innovation pour la Santé des Plantes, UFR Biosciences, Université Félix Houphouët-Boigny, Abidjan 22 BP 582, Côte d’Ivoire; 2 The Central and West African Virus Epidemiology (WAVE) for Food Security Program, Pôle Scientifique et D’innovation, Université Félix Houphouët-Boigny, Bingerville 22 BP 582, Côte d’Ivoire; 3 UFR Biosciences, Université Félix Houphouët-Boigny, Abidjan 22 BP 582, Côte d’Ivoire

**Keywords:** yam, YMV, YMMV, badnaviruses, CMV, distribution, RT-PCR, molecular characterisation, RCA-MinION

## Abstract

Yam (*Dioscorea* spp.) is a major staple food, contributing significantly to food security and income generation for millions of people worldwide. In 2019, surveys were conducted across the seven agro-ecological zones (AEZs) of Côte d’Ivoire, the third highest producer of yam globally, to ascertain the current status of viral diseases. In the 324 fields surveyed, a total of 1242 yam leaf samples were collected and tested for the presence of *Potyvirus yamtesselati* (yam mosaic virus, YMV), *Potyvirus yamplacidum* (yam mild mosaic virus, YMMV), *Cucumovirus CMV* (cucumber mosaic virus, CMV), and the badnaviruses using PCR, RT-PCR, and RCA followed by Sanger or MinION sequencing. The incidence of yam viral disease varied across the AEZs, with the lowest mean incidence observed in yam farms within the AEZ VII (71.95%) and the highest in AEZ V (88.15%). Viral disease symptom severity was moderate across the country, with more severe symptoms identified in AEZs II and VI. The virus screening revealed a potyvirus detection rate of 35.83% in all the AEZs. YMMV infection (25.12%) is the most prevalent in the samples, followed by YMV infection (15.61%). RCA-MinION sequencing revealed the presence of badnaviruses belonging to the T15 episomal groups K8, K9, and K5. Also, the use of this technique enabled the amplification and sequencing of four full-length episomal badnaviruses, namely Dioscorea bacilliform AL virus in group K8 and Dioscorea bacilliform RT virus in group K5. CMV was not detected in all the samples. It is noteworthy that 22.13% of mixed infections were detected in asymptomatic samples. This study revealed the first occurrence of YMMV in all the AEZs of Côte d’Ivoire. Of the yam species, *Dioscorea alata* was more widespread (78.03%) than *Dioscorea cayenensis-rotundata* (21.92%) in the visited fields. Also, *D. alata* had a highest incidence of YMMV (23.67%) infection than *Dioscorea cayenensis-rotundata*, while *D. cayenensis-rotundata* registered the highest incidence of YMV (15.84%) infection compared to *D. alata*. Phylogenetic analysis of representative of the various viruses detected in the country revealed that the sequences have high diversity for each virus species. This study revealed that viruses infecting yam are widespread and occur in mixed infection, which poses a real threat to yam production in Côte d’Ivoire.

## 1. Introduction

Yam (*Dioscorea* spp.) is a multi-species monocotyledonous tuber crop widely cultivated in Africa, Asia, South America, the Caribbean, and the South Pacific islands [1]. Globally, it ranks as the fourth most important root and tuber crop after potato, cassava, and sweet potato, contributing about 10% of total root and tuber production [2]. Yam is an important source of food and income for millions of people in tropical and subtropical regions, particularly in West and Central Africa, where at least 60 million people depend on it for their survival [3]. West Africa accounts for 95% of global yam production, with Nigeria, Ghana, Côte d’Ivoire, Benin, and Togo being the largest producers [4]. In Côte d’Ivoire, yam ranks first among agricultural crops, with an estimated production of 7,967,113.65 tonnes in 2023, and second in gross production value ($2.10 billion), next only to cocoa beans ($3.49 billion) [4]. Yam’s potential as a source of food is attributed to its high nutritional contents, including starch, sugars, dietary fibre, important minerals and vitamins, and higher dietary protein compared to other root and tuber crops, such as cassava. Nutritional composition varies significantly among species [5].

Yam also plays an important role in dietary diversification, and the peels are used as animal feed. Yam offers health benefits due to its potential medicinal applications in the treatment of some inflammatory diseases [5]. Beyond nutrition, yam holds economic, social, cultural, and religious significance in West Africa [6]. Its storability gives it an important role in food security, bridging periods of scarcity before harvest. In many communities, stored yam tubers are considered a form of wealth [2].

Despite its importance, yam remains an orphan crop and receives little research investment for agronomical improvement [5,7]. Furthermore, the crop is threatened by pests and various diseases, including those of viral aetiology. Since yam is clonally propagated from cuttings or whole tubers, viruses persist across seasons and spread through infected planting material [7]. Several species of *Dioscorea*-infecting potexviruses and macluraviruses have been reported, whereas in West Africa, the most common viruses are *Potyvirus yamtesselati* (yam mosaic virus, YMV), *Potyvirus yamplacidum* (yam mild mosaic virus, YMMV), *Cucumovirus CMV* (cucumber mosaic virus, CMV), and several badnaviral species [8,9,10,11,12,13].

Yam diseases caused by viral pathogens are among the most important, as they are known to reduce tuber yields by up to 40% [14,15]. Yam viruses cause an uneven loss of photosynthetic activity and often higher rates of chloroplast senescence, reducing sugar production and starch storage. As a result, tuber yield and quality are significantly reduced [16], and the exchange of planting material between farmers and across countries is a major means of virus propagation. YMV is the most widespread and damaging virus, especially in West Africa, the Caribbean, and the West Indies [14,17], while YMMV is the second most common yam virus, particularly in *D. alata*, often co-occurring with YMV [18]. DBVs are globally distributed and frequently symptomless, complicating detection and control [7]. The term DBV refers to *Dioscorea bacilliform viruses*, a diverse group of *badnaviruses* infecting yams (*Dioscorea* spp.), and the members possess circular double-stranded DNA genomes. At least 15 groups of DBVs have been identified. Further, the genome of the African yam, *Dioscorea cayenensis-rotundata,* has been demonstrated to host endogenous DBV sequences (eDBVs) originating from integrated badnavirus DNA in four distinct badnavirus groups (K5, K8, K9, and U12). Eight episomal DBV species have been identified in yams, and some species are still unknown [1,8]. Meanwhile, CMV, though less prevalent, is increasingly reported in mixed infections, adding to the overall disease burden on yam crops [19]. Yam mosaic virus was first described in Côte d’Ivoire by Thouvenel and Fauquet in 1977 [20]. Seka et al. [11] reported YMV and CMV in Bouaké and Toumodi, two yam-growing localities of Côte d’Ivoire. Bouaké and Toumodi had average YMV and CMV incidences of 88% and 95% and 10.85% and 16.28%, respectively, with mixed virus infections in 4.10% of the samples. Also, Toualy et al. [12] assessed 79 fields in the main yam-growing areas in Cte d’Ivoire and reported the presence of YMV, CMV, and yam-infecting badnaviruses, respectively, in 36%, 1.5%, and 39.1% of the samples tested. In addition to these three viruses, Bakayoko et al. [13] also detected potexvirus (family *Alphaflexiviridae*) and Dioscorea mosaic-associated virus (DMaV, family *Secoviridae*) in the national agricultural research centre (Centre National de Recherche Agronomique, CNRA)’s yam accessions collection. The DMaV genome is composed of two positive-sense RNA molecules and is from the genus *Sadwavirus* of the family *Secoviridae.* Effective management of yam viral diseases in Côte d’Ivoire requires updated information on the incidence, severity, diversity, and distribution of various virus species across all the agro-ecological zones, as well as their association with cultivated yam species. Such data are essential for developing effective control strategies. Therefore, this study aims to fill this knowledge gap by providing a comprehensive epidemiological assessment of yam viruses in the country.

## 2. Materials and Methods

### 2.1. Study Areas and Field Surveys

**Figure 1:**
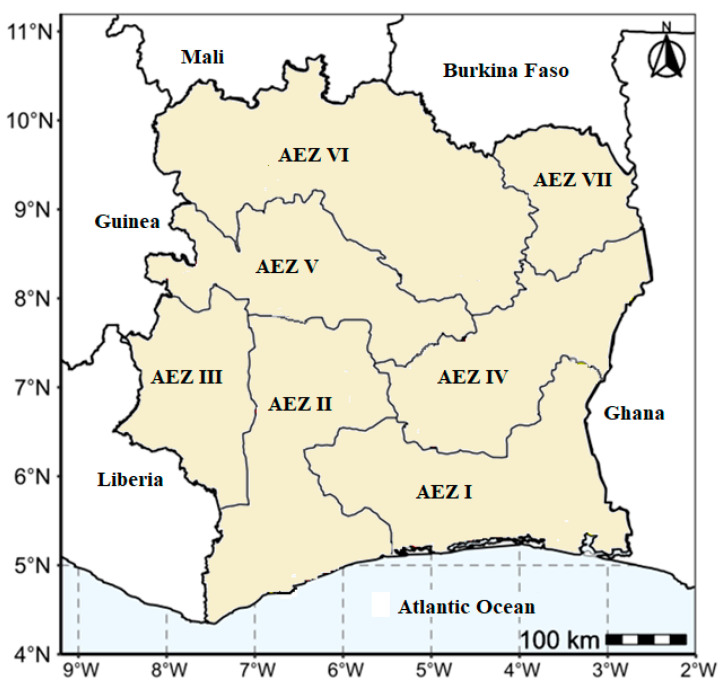
Locations of the six agroecological zones surveyed.

The study was conducted across the seven agro-ecological zones of Côte d’Ivoire (Figure 1). Field surveys were carried out in 2019, between July and September, in 33 localities across the country. In each locality, 7–15 fields were evaluated, with a minimum distance of 5 km between fields. The epidemiological assessment was performed on plants aged 3 to 6 months. The number of fields sampled within a locality depended on the density of yam fields in the locality.

#### 2.1.1. Disease Assessment and Determination of Epidemiological Parameters on *Dioscorea alata*, *Dioscorea cayenensis-rotundata*, and *Dioscorea bulbifera*

A total of 30 plants, randomly selected along two diagonals to form an “X” pattern, were assessed in each of the fields visited. Yam disease incidence (I) was calculated using the following formula:

I (%) = (No. of diseased-looking plants/Total no. of plants assessed) × 100(1)

Each selected plant was visually assessed for the presence or absence of viral disease symptoms (leaf mosaic, mottling, vein banding, bleaching, and stunted growth). Disease severity was scored using a scale of 1–5 established by the International Institute for Tropical Agriculture [21] (Table 1), where 1 represents the absence of disease symptoms and 5 is attributed to the most severe symptoms.

Mean severity (Sm) per field was calculated using the following formula:

Sm = Σ scores of diseased plants/Total number of diseased plants(2)

#### 2.1.2. Yam Leaf Sampling

In each yam field visited, fresh leaves with no symptoms, mild, severe, and very severe symptoms, such as mosaic, mottling, vein banding, puckering, and curling of leaves, were collected for virus detection. The number of samples collected per field depended on the species of yam and the symptoms observed. Two to four leaves were collected from each plant and stored in a tube as one sample. Leaves were preserved in 50 mL tubes containing 40 mL of 2% Cethyltrimethyl Ammoniac Bromide (CTAB) buffer and kept in a cool box during the surveys before being transported to the laboratory, where they were stored at 4 °C. The following information was recorded for each sample collected: yam species, location, sample status (with symptoms or not), symptom severity, and geographical coordinates.

### 2.2. Statistical Analyses

Data analysis was performed using the R software v. 3.6.1 (R Development Core Team) [22]. Normality of variables was determined using the Shapiro–Wilk test. If the variable was not normally distributed, the generalised linear model was used. The LSD test was used to analyse the differences between the means. Plots were generated using the ggplot2 v3.2.1 package [23]. The distribution maps for disease incidence, disease severity, and detected viruses were produced using the R software v. 3.6.1 (R Development Core Team) [22].

### 2.3. Molecular Detection of Yam Viruses

Total DNA was isolated from each sample using the Cethyltrimethyl Ammoniac Bromide (CTAB) method [24] supplemented with 1% sodium sulphite (Na_2_SO_3_) and 2% polyvinylpyrrolidone (PVP). Total RNA was also extracted using the CTAB protocol [24], which was modified to include a precipitation step using 4 M lithium chloride.

To detect RNA viruses (YMV, YMMV, and CMV), complementary DNA (cDNA) was synthesised via reverse transcription (RT) of extracted total RNA. Thus, the RT was performed in a mix containing 5 µM of oligo dT primer (NEB) and Random primers (NEB, Ipswich, MA, USA), 0.5 mM of dNTPs (NEB, Ipswich, MA, USA), 1× M-MuLV buffer (NEB, Ipswich, MA, USA), 10 U/µL of M-MuLV_RT (NEB, Ipswich, MA, USA), and 1 U/µL of RNase Inhibitor (NEB, Ipswich, MA, USA) in a total volume of 20 µL and submitted to the following programme: 65 °C for 5 min, 42 °C for 1 h, and 65 °C for 20 min. Note that the heating step at 65 °C was performed prior to the addition of the reverse transcriptase enzyme. The cDNA was then used for PCR as follows: a mix of 1× Colourless GoTaq reaction buffer (Promega, Madison, WI, USA), 0.625 U GoTaq polymerase (Promega, Madison, WI, USA), 0.2 μM of each primer (Eurogentec, Liège, Belgium), 0.2 mM dNTP (NEB), and 1.5 mM MgCl_2_ (Promega, Madison, WI, USA). The reaction volume was 25 μL, with 23 μL of the reaction mixture and 2 μL of 1:10 diluted cDNA. The reaction consisted of an initial denaturation step at 95 °C for 5 min, 35 cycles of 95 °C for 30 s, 55 °C for 30 s, 72 °C for 1 min, and a final extension of 72 °C for 10 min.

To obtain the coat protein (CP) genes from YMV- and YMMV-positive samples for sequencing, CP-specific primers were used alongside the same PCR mix described above, but with a different thermocycling programme: an initial denaturation step at 95 °C for 5 min; 35 cycles of 95 °C for 30 s, 55 °C for 1 min, and 72 °C for 1 min; and a final extension of 72 °C for 10 min. For the PCR amplification of the partial RT-RNase H gene, the reaction mixture consisted of 1× Colourless GoTaq reaction buffer (Promega, Madison, WI, USA), 0.625 U GoTaq polymerase (Promega, Madison, WI, USA), 0.4 μM of each primer (Eurogentec, Liège, Belgium), 0.2 mM of dNTP (NEB, Ipswich, MA, USA), and 1.5 mM of MgCl_2_ (Promega, Madison, WI, USA). The reaction volume was 25 μL, with 23 μL of the reaction mixture and 2 µL of 1:10 diluted DNA. The PCR programme for badnavirus sequences detection involved an initial denaturation at 94 °C for 4 min; 40 cycles of 94 °C for 30 s, 50 °C for 30 s, and 72 °C for 30 s; and a final extension at 72 °C for 5 min. The PCR/RT-PCR product (10 µL) was electrophoresed on 1% agarose gel. Then, the gel was stained with ethidium bromide and visualised under UV light using a gel imager (UV Transilluminator-26, VWR, Philadelphia, PA, USA). Details of the primers used for this work are provided in Table 2.

### 2.4. Sanger Sequencing and Phylogenetic Analysis

Positive and representative samples amplified using YMV F/R primers (50 samples) and YMMV F/R primers (20 samples) were selected to amplify the coat protein (CP) fragments of YMV and YMMV. The samples represented the seven agroecological zones (AEZs) and different yam species. These CP fragments were then sequenced. The 150 sequences of RT-RNase H domain were chosen according to the different agro-ecological zones (AEZs) and yam species. Purified partial reverse transcriptase ribonuclease H (RT-RNase H) domains of PCR positives were also sequenced by Genewiz (Leipzig, Saxony, Germany) using the Sanger method. These samples tested positive for badnaviruses. Purification was performed with phenol/chloroform prior to sequencing. The coat proteins of YMV and YMMV allowed for the study of their diversity. The portion of the RT-RNase H domain was sequenced to check if the DNA fragments amplified are typical members of *Badnavirus*. The obtained sequences were trimmed using Geneious Prime v. 2022.2.1. The sequence for each isolate was then used to perform a sequence similarity search in the GenBank databases using the BLAST programme (https://blast.ncbi.nlm.nih.gov/Blast.cgi) version 2.17.0, accessed on 31 May 2025, in order to check for the identity of viruses. Multiple sequence alignments were performed using the ClustalW component of MegaX [29], with sequences generated in this study and representative isolates of YMV, YMMV, and badnaviruses from Genbank. The maximum-likelihood (ML) method was used with MEGAX for YMV, YMMV, and badnaviruses with 1000 bootstrap replicates. Phylogenetic trees were constructed and visualised with MegaX, and edited using FigTree version 1.4.3 [30].

### 2.5. Rolling-Circle Amplification (RCA)–MinION Sequencing Strategy for the Amplification of Complete Genomes of Episomal Badnaviruses

Twenty samples, representative of the AEZs and yam species, were submitted for sequencing using the RCA-MinION strategy, in order to characterise potential badnaviral episomal genomes, as described by Otron et al. [31]. The samples were taken from *D. cayenensis-rotundata* and *D. alata*. RCA was performed using *Phi*29 DNA polymerase (Illustra TempliPhi Amplification, Marlborough, MA, USA), by mixing 2 µL of total plant DNA extract with 5 µL of Sample Buffer before incubation at 95 °C for 3 min. After cooling at room temperature, 0.2 µL of enzyme mix and 5 µL of Reaction Buffer were added before incubation at 30 °C for 20 h, followed by 10 min of polymerase deactivation at 65 °C. RCA products were then purified using Agencourt AMPure XP beads (Oxford Nanopore Technologies, Oxford, UK).

Library construction for MinION sequencing was carried out using the PCR Barcoding Kit (SQK-NBD114.96, Oxford Nanopore Technologies, Oxford, UK), following the manufacturer’s instructions, but using AMPure beads (Beckman Coulter, Brea, CA, USA) for the DNA purification steps. After the addition of the adapters, samples were pooled and cleaned. The eluted library was loaded onto a Flow Cell. Sequencing was performed using the MinION Mk1B device (MIN-101B, Oxford Nanopore Technologies, Oxford, UK). Super accurate basecalling was performed using Guppy (v6.3.2) [32], followed by demultiplexing and adapter removal. The demultiplexed reads were submitted to quality control, and the quality of the reads was investigated using NanoPlot v1.41.6 (https://github.com/wdecoster/NanoPlot?tab=readme-ov-file, accessed on 31 May 2025). The cleaned reads obtained were assembled using Flye 2.1 [33]. The contigs were polished using Medaka v1.7.2 (https://github.com/nanoporetech/medaka, accessed on 31 May 2025). The polished contigs were submitted to blastn online (https://blast.ncbi.nlm.nih.gov/Blast.cgi, accessed on 31 May 2025) against the non-redundant database (nr) using an E-value of 10^−4^ as the cut-off threshold value for significant hits.

## 3. Results

### 3.1. Incidence, Severity, and Distribution of Yam Virus Diseases Across the Agro-Ecological Zones

**Figure 2:**
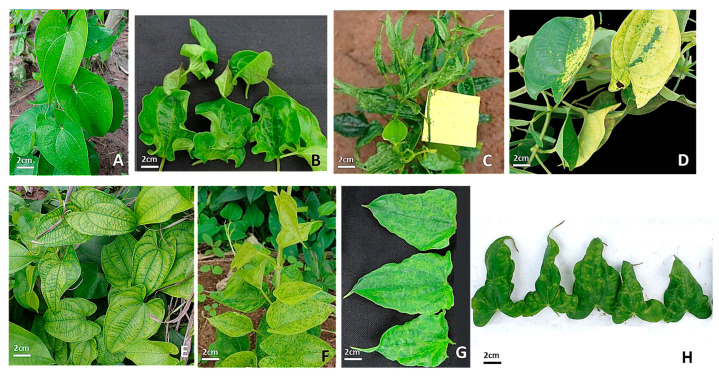
Symptoms of yam viral diseases observed in yam fields in Côte d’Ivoire: (**A**): healthy, (**B**): distortions, (**C**): shoe-string and stunting, (**D**): bleaching, (**E**): mottling and vein banding, (**F**): chlorotic mottle, (**G**): mosaic, (**H**): puckering. *Dioscorea alata* (**C**,**E**–**H**); *Dioscorea cayenensis-rotundata* (**A**,**B**,**D**).

**Figure 3:**
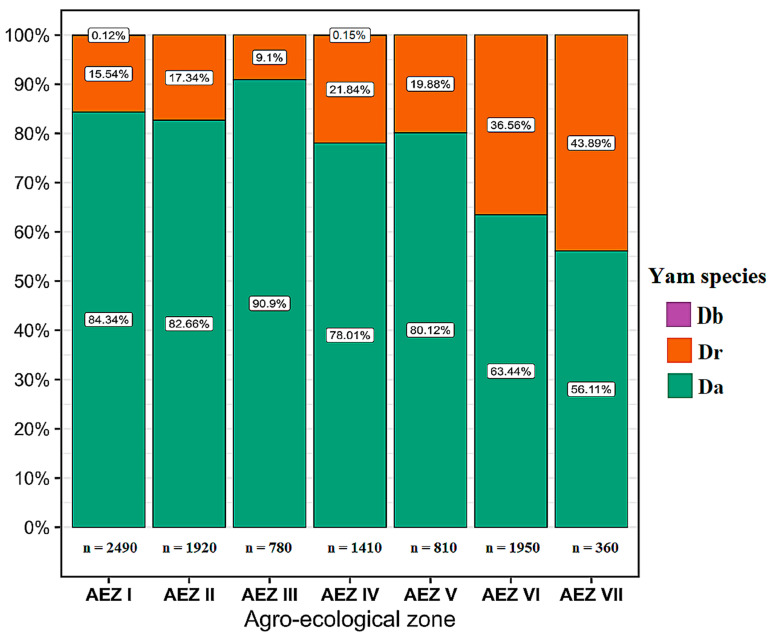
Repartition of *Dioscorea alata*, *Dioscorea cayenensis-rotundata,* and *Dioscorea bulbifera* fields assessed according to the Agro-ecological zones. Legend: Da: *Dioscorea alata*, Dr: *Dioscorea cayenensis-rotundata*, and Db: *Dioscorea bulbifera*.

During the surveys, several yam disease symptoms were observed in all 324 yam fields surveyed across the seven agro-ecological zones, including mosaic, mottling, vein banding, puckering, vein clearing, bleaching, chlorosis, shoestring, distortion, and stunting (Figure 2). In all the agro-ecological zones, most farmers cultivated *D. alata*, followed by *D. cayenensis-rotundata* and *D. bulbifera;* therefore, *D. alata* fields represented the largest proportion of fields assessed (Figure 3).

**Figure 4:**
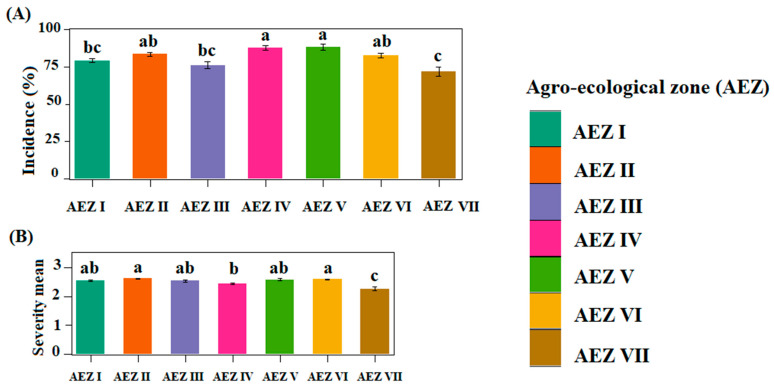
Epidemiological assessment of viral disease symptoms in yam fields surveyed in Côte d’Ivoire: (**A**) incidence of yam viral symptoms; (**B**) mean symptoms severity. Statistical significance was calculated using the LSD test at a 0.5 threshold (α = 0.05); the same letters are not significantly different between agro-ecological zones (AEZs).

A total of 1242 symptomatic and asymptomatic yam leaf samples were collected from the 324 fields assessed. The mean incidence of yam viral diseases ranged from 43.33% to 100% in the fields visited, with an overall mean of 81.43% (Figure 4A). Statistical analyses showed a significant difference (*p* < 0.05) in the mean incidence of yam viral diseases across the seven agro-ecological zones (AEZs). AEZ V had the highest mean incidence (88.15 ± 2.06), followed by AEZ IV (87.94 ± 1.56). These two zones are major yam-producing areas in the country. AEZ II and AEZ VI, two other major areas for yam production, followed, with mean incidences of 83.65 ± 1.34 and 82.67 ± 1.33, respectively. The lowest mean incidence was recorded in AEZ VII (Figure 4A). Our study also showed that viral disease incidence was higher in *D. cayenensis*-*rotundata* than in *D. alata*, despite *D. alata* fields being more widespread. The mean symptom severity was generally mild and varied from 2 to 3.14, with a mean of 2.51 across the country. The mean symptom severity between AEZs were significantly different (*p* < 0.05). The highest mean severity was recorded in AEZ II (2.62 ± 0.02), followed by AEZ VI (2.59 ± 0.02), while the lowest mean severity was registered in AEZ VII (2.27 ± 0.06; Figure 4B). There was no significant difference in disease severity between *D. alata* and *D. cayenensis*-*rotundata*.

**Figure 5:**
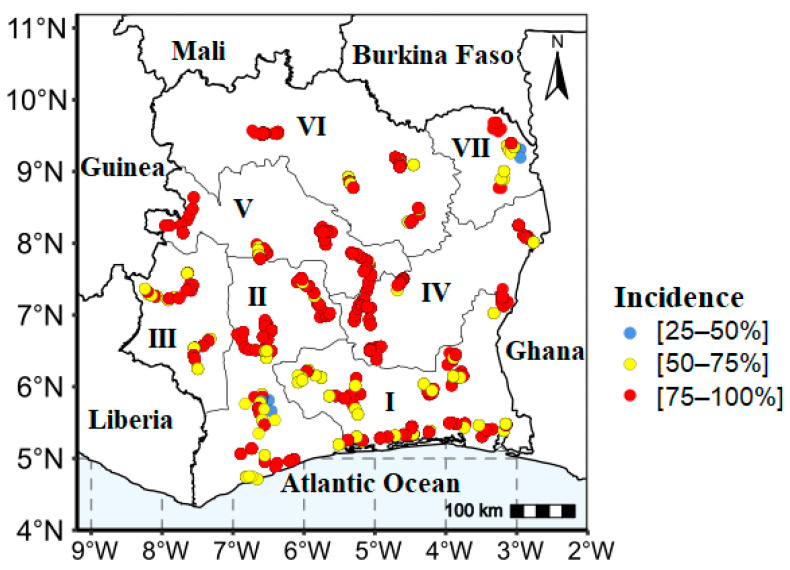
Yam viral disease symptom incidence in the seven agro-ecological zones in Côte d’Ivoire. Agro-ecological zones AEZs (I, II, III, IV, V, VI, VII).

The result shows the presence of yam viral disease symptoms in all agro-ecological zones of the country (Figure 5). High incidences (75–100%) were primarily widespread in the central and western regions of the country, while the southern region predominantly exhibited moderate incidences ranging from 25 to 75%.

### 3.2. Molecular Detection of YMV, YMMV, and CMV (RNA Viruses)

**Figure 6:**
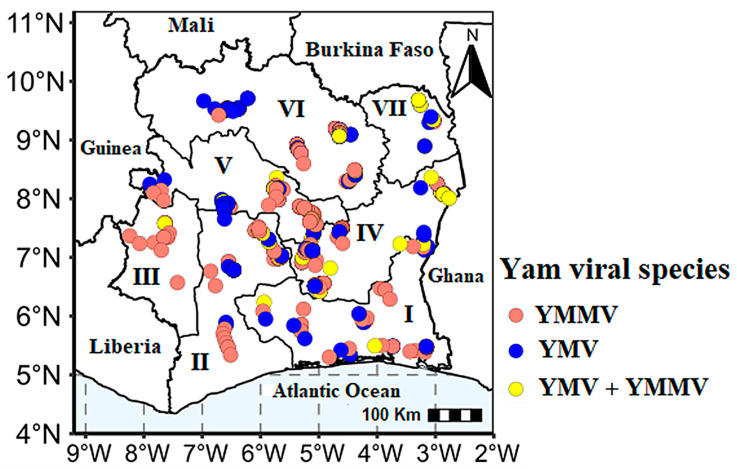
Distribution of YMV and YMMV, in single and mixed infections in yam farms across the seven agro-ecological zones (AEZs) in Côte d’Ivoire.

RT-PCR analyses detected the presence of yam mosaic virus and yam mild mosaic virus in the yam leaves collected throughout the country (Figure 6). In general, 35.13% (445/1242) of the collected sample tested positive for at least one virus. Cucumber mosaic virus (CMV) was not detected in our samples. Statistical analyses showed significant differences in the detection rate of the different viruses across the AEZ and mixed infection (Table 3).

It was also noted that 19.82% of the symptomatic samples analysed were negative for all the viruses tested for in this study, with AEZ I and II alone accounting for 14.12% of these symptomatic but PCR-negative samples. As for the asymptomatic samples, 28.51% were positive for YMV and YMMV, with YMMV single infection accounting for 58.96% (79/134) of positive cases, followed by 14.18% (19/134) cases of mixed infection.

Without species or strain discrimination, AEZs IV (100/179) and VI (99/170) showed the highest rate of positive samples to the potyviruses compared to the other zones. YMMV single infection (20.21%) was the most predominant in all seven zones, followed by YMV single infection (10.71%).

The incidence of YMMV was highest in AEZs IV (34.64%) and V (29.06%), followed by zone VI (24.12%), while zone VII showed the lowest level of infection (6.52%). The incidence of YMV was quite moderate across all the zones, except in AEZ III, where YMV was not detected in single infection but only in mixed infection. The highest rate of single YMV infection was observed in AEZ VI (25.29%), followed by AEZs IV (12.85%) and V (12.81%) (Table 3). The rate of mixed YMV and YMMV infection was 4.91%, and this infection is widespread in all the zones. YMV and YMMV mixed infection was particularly high in AEZs VII (13.04%), followed by AEZs VI (8.82%) and IV (8.38%). The lowest infection rate was reported in the AEZ III (1.08%) (Table 3).

### 3.3. Presence of YMV and YMMV in Distinct Yam Species

The interaction between viral species and yam species was found to be highly significant across the entire country, with a *p*-value of less than 0.05 (*p* = 0.003).

**Figure 7:**
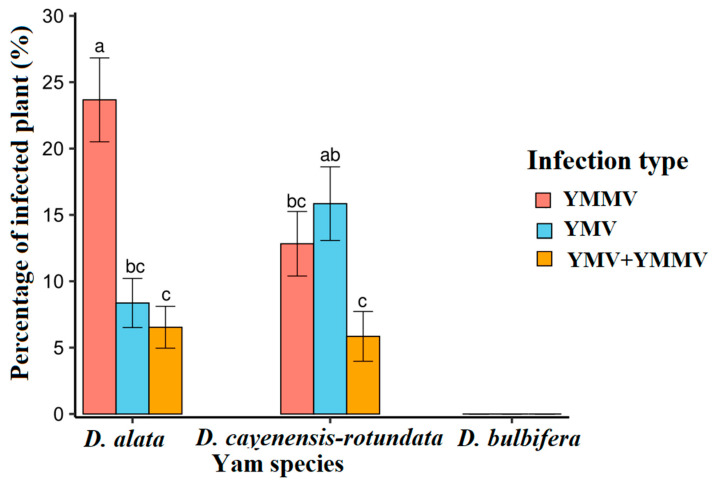
Proportion of infected plants according to the virus in each yam species. Statistical significance was calculated using the LSD test at a 0.5 threshold (α = 0.05). Percentages followed by the same letters are not significantly different.

YMV and YMMV were only detected in *D. alata* and *D. cayenensis-rotundata* but not in *D. bulbifera*. Also, YMMV was more prevalent in *D. alata* than *D. cayenensis-rotundata*, while YMV was more important in *D. cayenensis-rotundata* (Figure 7). In the two yam species, a statistically identical proportion of mixed infection was observed.

### 3.4. Phylogenetic Analysis of Viral Sequences of YMV and YMMV

A total of 24 partial sequences were obtained from YMV (20) and YMMV (04).

**Figure 8:**
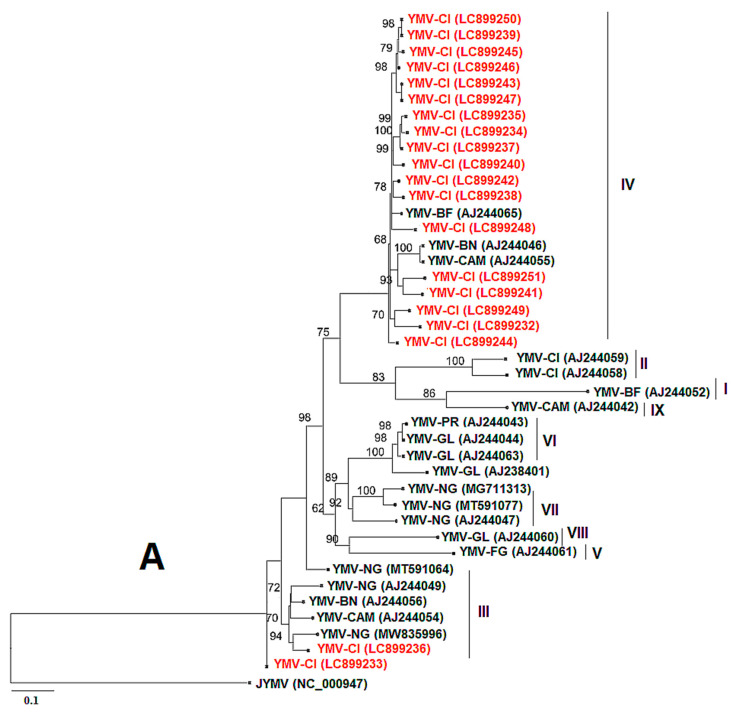

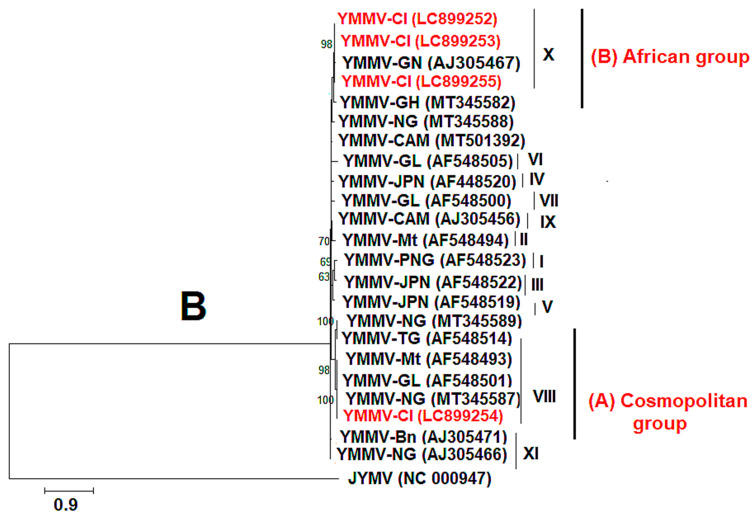
Maximum-likelihood phylogenetic tree indicating the relationships of partial yam mosaic virus (YMV) and yam mild mosaic virus (YMMV) nucleotide sequences from this study and diverse representative isolates of the three viruses. (**A**) The tree is based on the coat protein (CP) of yam mosaic virus and rooted using Japanese yam mosaic virus (GenBank accession NC 000947) as an outgroup. The different groups are made following the classification of Bousalem et al. [34]. (**B**) The tree is based on the coat protein (CP) of yam mild mosaic virus and rooted using Japanese yam mosaic virus (GenBank accession NC 000947) as an outgroup. The groups are formed following the classification of Bousalem et al. [35] and Nkere et al. [18]. The sequences from GenBank are coloured black, and the sequences from this study are in red. Bootstrap analysis was performed with 1000 replicates, and the horizontal scale indicates the genetic distance.

For YMV, coat protein (CP) sequences of approximately 906 bp in length were obtained using the YMV CP primer pair. The sequences with the following accession numbers were isolated from *D. cayenensis–rotundata* (LC899233, LC899243, LC899245, LC899246, LC899249, and LC899250), while the other sequences originated from *D. alata* (LC899232, LC899234, LC899235, LC899236, LC899237, LC899238, LC899239, LC899240, LC899241, LC899242, LC899244, LC899247, LC899248, and LC899251). The YMV CP from this study showed 94.83% to 98.50% nucleotide sequence identity with YMV sequences from Burkina Faso (AJ244065.1), Benin (AJ244046.1), Nigeria (MZ670956.1, MZ670987.1, MT542863.1, MZ670884.1, MZ671001.1, and MT501420.1), and Cameroon (AJ244055.1). Figure 8A shows that our sequences are mainly divided into two phylogenetic groups (IV and III), based on the classification of Bousalem et al. [34], and clustered mainly with sequences from West Africa. The nucleotide sequences of YMV CP generated in this study have been deposited in the GenBank database under accession numbers LC899232 to LC899251. For YMMV, coat protein (CP) sequences of approximately 798 bp in length were obtained using the YMMV CP primer pair. BLASTn search in the GenBank database showed that the CP sequences of YMMV infecting only *D. alata* leaves in Côte d’Ivoire shared a high nucleotide identity (89.41% to 99.33%) with YMMV isolates from Ghana (MT345582.1), Nigeria (MT345588.1, MT345587.1, MT345585.1, OM471978.1), Cameroon (MT501392.1), and Martinique (AF548493.1). The nucleotide sequences of YMMV CP generated in this study have been deposited in the GenBank database under accession numbers LC899252 to LC899255. The phylogenetic tree presents the relationship between these sequences and those from this study (Figure 8B). It shows that our sequences fall into two phylogenetic groups (cosmopolitan group (A) and African group (B)) based on the classification of Bousalem et al. [35] and Nkere et al. [18].

### 3.5. PCR Amplification of Badnavirus Sequences (DNA Viruses)

In total, 513 of the 1242 yam samples (41.30%) tested were considered PCR-positive to the amplification using the primers Badna FP/RP. The sequencing of representative (according to AEZs and yam species) (150) purified bands found as PCR-positive allows us to confirm the presence of the genus *Badnavirus*. In fact, the sequences showed 85.71% to 94% nucleotide identity for other badnavirus sequences from Côte d’Ivoire (MN477403.1, MN477394.1, MN477390.1, MN477405.1), Nigeria (AM944572.1, KY555563.1, AM944573.1, KX008584.1), Guadeloupe (KF829986.1), Benin (AM944584.1, AM944588.1, AM944583.1), Ghana (AM072668.1, AM944580.1, AM072666.1, AM944577.1), and Togo (AM944582.1, AM944580.1). However, PCR alone cannot confirm the episomal status; then, efficiently perform the exploration of the full diversity of badnaviruses and amplify putative complete genomes of episomal virus. The RCA, combined with MinION, allowed for a better understanding of the diversity, as it permits deep sequencing.

### 3.6. Analysis of Partial Sequences and Full-Length Genomes of Episomal Badnaviruses and Their Phylogenetic Relationships

After RCA-MinION sequencing, twelve partial episomal badnavirus sequences were obtained. An analysis of the RT–RNase H domain showed an 85% to 99% nucleotide identity for previously reported sequences, mainly from Côte d’Ivoire (MN477403, MN477404, MN477394), Nigeria (KX008572, KX008584, NC_076205), and Guadeloupe (KX430257). A phylogenetic analysis revealed that these sequences clustered into four monophyletic groups: K8 (five sequences), K5 (three sequences), K9 (two sequences), and T15 (two sequences) (Table 4). This approach broadens the characterisation of badnavirus diversity in yam in Côte d’Ivoire. The nucleotide sequences of partial RT-RNase H generated from the RCA-MinION were deposited in the GenBank database under accession numbers LC899260 to LC899271.

Four complete genomes of episomal badnaviruses had been amplified. A complete sequence analysis revealed the presence of two genomes belonging to the species *Badnavirus alphadioscoreae* (Accession numbers PX314964 and PX314965), as well as two genomes of *Badnavirus zetadioscorea* (Accession numbers PX314966 and PX314967). Sequence annotation showed that the complete genomes contained all three open-reading frames (ORFs) typical of badnaviruses.

Dioscorea bacilliform AL virus (DBALV)

The two complete genomes (DBALV-CI2019-Zue (PX314964) and DBALV-CI2019-Touba (PX314965)) were obtained from *D. cayenensis-rotundata* and *D. alata,* respectively. The sequences were 7609 bp and 7405 bp in length, with GC contents of 43.9% and 43.5%, respectively. Annotation of the complete genomes resulted in the identification of the expected open reading frames (ORFs), with ORF 1 being 432 bp in length, ORF 2 being 378 bp in length in both genomes. As for ORF 3, lengths of 5769 bp and 5697 bp were recorded. This ORF 3 contained conserved domains corresponding to the movement protein (MP), capsid protein zinc-finger domain (CP and Zn knuckle), reverse transcriptase (RT), and RNase H. Conserved motifs also included the initiator, methionine tRNA sequence (tRNAmet site), located in the intergenic region at position 1–18, indicating the starting point of the viral genomes. The tRNAMet-binding-site sequences of DBALV-CI2019-Zue 5 and DBALV-CI2019-Touba (5′-TGGTATCAGAGCTTGGTT-3′) share 16 of 18 nucleotides that match the consensus of the plant tRNAMet-binding site (3′-ACCAUAGU CUCGGUCCAA-5′). In addition, a TATA-box (TATATAA) and polyadenylation signal, analogous to the 35S promoter of cauliflower mosaic virus (CaMV), were also identified in the intergenic region (IR). A BLASTn analysis of the full-length genome sequences confirmed that DBALV-CI2019-Zue 5 and DBALV-CI2019-Touba were closely related to previously published DBALV complete genomes (GenBank accessions NC_038381 and MH404166), with nucleotide sequence identities of 91.89 to 91.26%, respectively.

Dioscorea bacilliform RT virus 3(DBRTV3)

The two complete episomal sequences (DBRTV3-CI2019-Dabak (PX314966) and DBRTV3-CI2019-Man (PX314967)) were generated from *D. cayenensis-rotundata.* The sequence lengths were 7518 bp and 7507 bp, and they possessed GC contents of 44.2% and 43.5%, respectively. Both genomes shared the same lengths for ORF 1 (432 bp) and ORF 2 (390 bp), while ORF 3 was measured at 5733 bp in DBRTV3-CI2019-Dbak and 5515 bp in DBRTV3-CI2019-Man, and also presented the expected conserved domains. The tRNAMet-binding-site sequences of DBRTV3-CI2019-Dabak and DBRTV3-CI2019-Man also share 16 of 18 nucleotides identical to the consensus sequence. Each genome contained a TATA-box (TATATAA) located upstream of the tRNAMet-binding site and a poly(A) signal within the intergenic region (IR). DBRTV3-CI2019-Dabak and DBRTV3-CI2019-Man shared the highest nucleotide similarity with DBRTV3 isolate NC_076205, with identity values of 89.02% and 89.41%, respectively.

**Figure 9:**
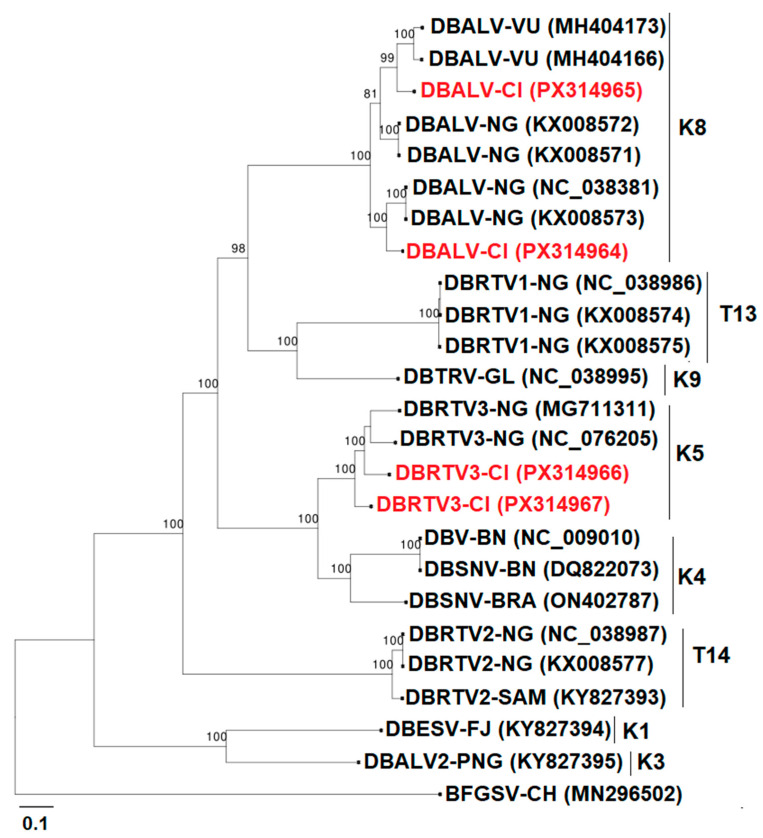
Maximum-likelihood phylogenetic tree indicating the relationships between four complete episomal badnaviruses and diverse representatives of the badnaviruses. The sequences fell into two already described monophyletic species groups (K5 and K8). K8 and K5 include putative endogenous sequences and episomal badnaviruses. The tree is rooted using Banana streak GF virus (GenBank accession MN296502) as an outgroup; the different groups are formed following the classification of Bömer et al. [36], Kenyon et al. [37], and Umber et al. [38]. The groups (K1–K11), U12, and T13–15 are represented. The sequences from GenBank are coloured black, and the sequences from this study are in red. Bootstrap analysis was performed with 1000 replicates, and the horizontal scale indicates the genetic distance.

Phylogenetic analysis of the complete episomal genomes revealed that they clustered in monophyletic groups K5 and K8, showing a close relationship with previously published complete genome sequences (Figure 9).

## 4. Discussion

The surveys conducted in Côte d’Ivoire provided information on the incidence, severity, and distribution of three viruses infecting yam in the seven agro-ecological zones. The average disease incidence in the field varied considerably from one AEZ to another and was particularly high in AEZs IV and V, which are located in the transition zone. This high incidence is probably due to the use of infected yam cuttings for planting year after year, which perpetuates the same virus(es) in new fields. In fact, farmers report using their own tubers or buying their planting material from the market. Neither farmers nor traders are aware of yam virus diseases, which contributes to their spread. The overall mean yam virus disease incidence of 81.44% obtained in this study is much higher than the 36.2% reported by Toualy et al. in 2014, 10 years ago [12]. The use of infected cuttings combined with the increase in yam cultivation over time could explain this higher incidence. In fact, from 2000 to 2023, the area of land under yam cultivation in Côte d’Ivoire increased from 829,595 to 1,499,028 ha to meet the needs of the population [4].

Overall, the average disease severity was moderate in all AEZs, with higher mean severities occurring in AEZ II and AEZ VI, which are located in the south and north of the country, respectively. Indeed, the spread of viruses over these geographical regions increases the risk of interactions with local virus reservoirs and the neighbouring countries’ viruses, which can lead to the emergence of more aggressive recombinant strains, as well as more complex and costly management approaches.

During the surveys, we observed symptoms of mosaic, mottling, vein banding, puckering, vein clearing, bleaching, chlorosis, shoestring, distortion, and stunting. The same symptoms were observed in samples from previous studies carried out in Nigeria, Ghana, Benin, and Côte d’Ivoire [9,11,12,13].

The occurrence of higher YMMV (25.12%) infection than YMV (15.61%) infection in Côte d’Ivoire is noteworthy because, in most previous studies, YMV was the unique prevalent potyvirus in the country [12,13,39]. This observed change in trend may need to be further investigated, with a view to ascertaining the extent of yield losses associated with YMMV infection and to inform management recommendations and the appropriate response level required. The occurrence of single YMMV infection was predominant, and AEZ IV had the highest rate of single YMMV infection (34.64%). This situation is serious because this zone (IV) is a focal point for yam production in Côte d’Ivoire and is located on the border with Ghana, the second-largest yam-producing country globally. This emergence of high levels of YMMV infection in yam-growing communities was also reported in Nigeria by Asala et al. [40] in surveys conducted in 2010, where YMMV (21.4%) was more prevalent than YMV (9.2%). Our results differ from those of Bakayoko et al. [13], who reported that YMMV was not detected in yam accessions from Bouaké, and this could be explained by the absence of *D. alata* accessions in their study, which is the most prevalent for YMMV. Moreover, this may also be attributed to the low prevalence of YMMV in *D. cayenensis*-*rotundata* compared to *D. alata* [35].

Mixed infection of YMV and YMMV was widespread across the AEZs. This fact is common among vegetatively propagated crops, and the occurrence of co-infection of viruses in yam has been reported in Nigeria, Ghana, Benin, Togo, and Côte d’Ivoire [9,10,11,12,13,41]. Co-infections involving several viruses can exacerbate the severity of symptoms and increase yield losses. Moreover, mixed infections of multiple virus species and strains in vegetatively propagated crops, such as yams, provide conditions that favour the emergence of new strains via recombination [42]. Furthermore, Diouf et al. [43] showed the same results in their study on the epidemiology of yam viruses in Guadeloupe. Interestingly, 19.82% of the samples showing apparent symptoms of virus infection were negative for the viruses tested. This could potentially indicate the presence of either novel unreported viruses or other viruses not tested in this study. Furthermore, symptoms might potentially be the result of abiotic variables, such as senescence and nutritional disorders, which produce symptoms similar to those of viruses, as described by other authors [13,19]. The occurrence of YMV and YMMV in 28.51% of symptomless samples may indicate that the plants are either tolerant to the viruses or that viruses are in a latent state within the plants, thus not exhibiting obvious symptoms. Therefore, the use of visual symptom assessment for the certification of yam viral diseases is not advisable. The use of laboratory testing for the certification of yam-planting materials is a crucial prerequisite for limiting the spread of viruses in yam fields.

We observed a significant interaction between yam virus infection type and yam species in this study. This indicates that the yam species grown in each AEZ may influence the associated viruses. *D. alata* had a far higher YMMV infection rate than *D. cayenensis-rotundata*, likely because of its high virus sensitivity. Similar to Odu et al. [44], we found that *D. alata* was commonly infected with YMMV in Africa. Moreover, according to Bousalem et al. [35], *Dioscorea alata* had a significantly higher prevalence of YMMV than *D. cayenensis-rotundata* (11.2% and 3.6% of infected samples, respectively) in samples from Guadeloupe, Martinique, and French Guyana. The sensitivity of *D. cayenensis-rotundata* to YMV explains why this species is commonly infected [13]. Similarly, Bakayoko et al. [13] found this virus in all *D. cayenensis-rotundata* accessions in Bouake. Also, Eni et al. [9] showed an incidence of YMV three times higher in *D. cayenensis*-*rotundata* compared to *D. alata*.

Mixed infections are present in both yam species. The replication of two different potyviruses within the same living host would most likely exert stress/pressure on the host and, in this case, interfere with tuber yield. This may ultimately impact yam production in Côte d’Ivoire, given that *D. alata* is the most cultivated yam species in the country, followed by *D. cayenensis-rotundata*. In contrast, CMV was not detected in our tested samples. However, Toualy et al. [12] reported a low CMV prevalence of 1.5%. This difference in results could be due to the use of ELISA by Toualy et al. in their study. In fact, Séka et al. [11] also found CMV in Bouaké and Toumodi using ELISA.

The majority of YMV isolates from this work were more closely related to isolates from Burkina Faso, Benin, Cameroon, Nigeria, and Côte d’Ivoire according to phylogenetic analysis. This is most likely a result of plant material being exchanged between Côte d’Ivoire and these other nations. Three of the YMMV sequences from this study belong to the African group (group B) according to Nkere et al. [18] and are closely related to isolates from Ghana and Nigeria, with only one of our sequences belonging to group A, which represents the cosmopolitan group. The cosmopolitan group (A) gathered essential sequences of group VIII, which contains isolates from Togo, Japan, Martinique, and Guadeloupe. This cosmopolitan clustering pattern could reflect extensive international movement of infected planting material and/or evolutionary processes, such as recombination and shared selection pressures across regions.

At least fifteen groups of Dioscorea bacilliform viruses (DBVs), which belong to the genus *Badnavirus* in yam, have been identified so far [36,37,38,45], and four of the fifteen groups have been reported to contain some endogenous virus sequences, namely K5, K8, K9, and U12 [38,46,47,48]. Sanger sequencing of PCR-positive samples to the RT-RNase H domain confirmed the presence of badnavirus in the sequenced samples. The twelve sequences obtained by RCA-MinION cluster into four distinct monophyletic groups (K5, K8, K9, T15) and share 76–100% nucleotide identity with their nearest NCBI matches. The 100% identity value was obtained with another episomal badnavirus in the K8 species group, namely DBALV-[3RT] isolate (NC_038381). Notably, the highest identity to known endogenous DBV-like sequences is only 95%, which is lower than the high conservation (>98%) typically observed for integrated viral fragments [46]. In addition, RCA-MinION sequencing enabled the recovery of four complete circular genomes, providing direct molecular evidence of their episomal nature. Together, the phylogenetic placement, sequence diversity, and full-length genome circularity strongly support that the twelve sequences represent episomal badnavirus actively infecting yam plants in Côte d’Ivoire. The two K9 group sequences obtained by RCA-MinION, confirmed as episomal, represent the first documented detection of this badnavirus species in Africa. This finding extends the known geographical range of the K9 lineage and raises important questions about its introduction and potential impact on yam production. This suggests persistent circulation of potential episomal viral lineages in yam material in the germplasm and some fields of Côte d’Ivoire. Two complete genomes of Dioscorea bacilliform AL virus (PX314964 and PX314965) and two complete sequences of Dioscorea bacilliform RT virus (PX314966 and PX314967) were identified and belong to K8 and K5, respectively. Full episomal genome amplification suggests a strong indication of active badnavirus infection in the tested samples. These results showed the ability of RCA [36], combined with MinION sequencing, to recover complete viral genomes, providing reliable data. It should be noted that the badnavirus results concern only a small number of samples. It was not possible to determine the prevalence of badnaviruses using PCR in this study because yam contains endogenous badnavirus sequences (eDBVs), and antibodies for immunocapture-PCR were unavailable. PCR alone cannot reliably distinguish between endogenous and episomal viral genomes, so positive detections may not reflect active infections, making prevalence estimates unreliable. The obtained sequences will contribute to the design of specific primers to evaluate the prevalence and distribution of episomal viruses throughout the country and deepen our knowledge of badnaviruses.

## 5. Conclusions

This study reports the first detection of YMMV in the agro-ecological zones and the spread of mixed infection of YMV, YMMV, and badnaviruses in the yam fields of Côte d’Ivoire. Four monophyletic groups of badnaviruses exist in the country, namely K8, K9, K5, and T15. The sequence analysis of the detected viral species denotes the potential inter-country transmission of viruses between nations, most likely due to the exchange or movement of infected plants over porous land borders, particularly in West Africa. The rapid spread of viral diseases requires special attention from the authorities and the introduction of appropriate control measures and healthy planting material through tissue culture.

## Figures and Tables

**Table 1:** Description of symptom severity score of yam viral diseases.

Score	Associated Severity of Viral Symptoms
1	Symptomless plants
2	Plants presenting moderate symptoms (1–25% of the leaves)
3	Plants presenting severe symptoms (26–50% of the leaves)
4	Plants presenting very severe symptoms (51–75% of the leaves)
5	Severely attacked plants presenting >75% distorted or malformed leaves and/or with signs of dwarfism

**Table 2:** Primer pairs for the detection of yam mosaic virus (YMV), yam mild mosaic virus (YMMV), cucumber mosaic virus (CMV), and badnaviruses.

Primer’s Name	Primer Sequence (5′-3′)	Size (bp)	Target Region	References
CMV-F CMV-R	GCCGTAAGCTGGATGGACAA	500	RNA3	Wylie et al., 1993 [25]
	TATGATAAGAAGCTTGTTTCGCG			
YMV-F YMV-R	ATCCGGGATGTGGACAATGA	586	CP/3′UTR	Mumford and Seal, 1997 [26]
	TGGTCCTCCGCCACATCAAA			
YMMV-F YMMV-R	GGCACACATGCAAATGAARGC	259	CP/3′UTR	Mumford and Seal, 1997 [26]
	CACCAGTAGAGTGAACATAG			
YMMV CP-Bam FP YMMV CP-EcoRP	CAGAGAGGATCCGCAAGTAAGGAACAGACATTTG	798	CP	N’kere et al., 2020 [18] N’kere, 2015 [27]
	TTGATCGAATTCCTAGATATTGCGCACTCCAAGAAG			
YMV CP Bam-FP YMV Eco-RP	AGAGGATCCGCAGATACACAGCCAGATG	906	CP	N’kere, 2015 [27]
	ATCGAATTCCTACATACCTCTCATGCCCAAAAG			
Badna FP Badna RP	ATGCCITTYGGI ITIAARAAYGCICC	579	RT-RNase H	Yang et al., 2003 [28]
	CCAYTTRCAIACISCICCCCAICC			

**Table 3:** Occurrence of YMV and YMMV in single and mixed infection in yam fields in seven agro-ecological zones in Côte d’Ivoire.

AEZ	Sample (*n*)	Positive	YMV (%)	YMMV (%)	YMV + YMMV (%)
I	314	56	6.36 ^b^	10.19 ^c^	1.27 ^b^
II	237	64	6.33 ^b^	16.87 ^bc^	3.79 ^ab^
III	93	15	0 ^b^	15.05 ^bc^	1.08 ^b^
IV	179	100	12.85 ^b^	34.64 ^a^	8.38 ^a^
V	203	96	12.81 ^b^	29.06 ^a^	5.42 ^ab^
VI	170	99	25.29 ^a^	24.12 ^ab^	8.82 ^a^
VII	46	15	13.04 ^ab^	6.52 ^c^	13.04 ^a^
Total	1242	445	10.71	20.21	4.91

Note: YMV, yam mosaic virus; YMMV, yam mild mosaic virus. Statistical significance was calculated using the LSD test at a 0.5 threshold (α = 0.05). Percentages followed by the same letters are not significantly different between agro-ecological zones (AEZs).

**Table 4:** RCA-derived sequences and BLAST analysis of partial RT-RNase H (528 bp) coding regions.

Plant Accession	Yam Species	Accession	Size	NCBI Nearest Match	Identity (%)	Species Groups
B4C22	Da	LC899260	528	AM944572	95.45	K8
B4C73	Da	LC899261	528	AM944572	95.45	K8
B8C125	Dr	LC899262	528	NC 038381	100	K8
B19C220	Dr	LC899263	528	KF829986	95.64	K8
B3C22	Da	LC899264	528	MN477403	85.07	K8
B13C176	Dr	LC899267	528	MN477407	96.4	K5
B14C17	Dr	LC899268	528	MG711311	93.18	K5
B1C261	Dr	LC899269	528	AM072659	91.48	K5
B10C209	Dr	LC899265	528	KX008584	93.18	T15
B2C159	Da	LC899266	528	MN477394	92.60	T15
B4C236	Da	LC899270	528	AM503361	85.45	K9
B10C186	Da	LC899271	529	KF829957	76.42	K9

## Data Availability

The datasets generated during and/or analysed during the current study are available from the corresponding author on reasonable request. The complete genome sequences of badnavirus isolates obtained from Nanopore sequencing are available on GenBank under the accession numbers PX314964-PX314967. The partial sequences generated in this study are already deposited in GenBank under accession numbers from LC899232 to LC899271.
